# Comparison of anterior transthoracic debridement and fusion with posterior transpedicular debridement and fusion in the treatment of mid-thoracic spinal tuberculosis in adults

**DOI:** 10.1186/s12891-019-2945-x

**Published:** 2019-11-27

**Authors:** Weiwei Li, Zheng Liu, Xiao Xiao, Zhen Zhang, Xiyang Wang

**Affiliations:** 1 Department of Spine Surgery, Xiangya Hospital, Central South University, 87#Xiangya Road, Changsha, 410008 Hunan China; 2 Hunan Engineering Laboratory of Advanced Artificial Osteo-materials, Xiangya Hospital, Central South University, 87#Xiangya Road, Changsha, 410008 Hunan China; 3 Department of Orthopedic, Shaanxi Provincial People’s Hospital, Xi’an, 710068 Shaanxi China

**Keywords:** Thoracic, Spinal tuberculosis, Transthoracic, Transpedicular, Anterior, Posterior

## Abstract

**Background:**

The surgical procedures for mid-thoracic spinal tuberculosis mainly include anterior transthoracic debridement and fusion and posterior transpedicular debridement and fusion. Until now, the surgical choice is still controversial. This study aims to compare the clinical efficacy of anterior transthoracic debridement and fusion with posterior transpedicular debridement and fusion in the treatment of mid-thoracic (T5–9) spinal tuberculosis in adult patients.

**Methods:**

Eighty-seven cases with mid-thoracic spinal tuberculosis were treated with anterior transthoracic debridement and fusion (Group A, *n* = 39) and posterior transpedicular debridement and fusion (Group B, *n* = 48) from January 2007 to June 2014. Parameters including the operation time, blood loss, time of ESR and CRP decreasing to the normal level, time of abscess disappearance, time of bone graft fusion, rate of surgical complications, Visual Analog Scale (VAS) score, kyphosis angle and SF-36 scale were compared between two groups to evaluate their therapeutic effects.

**Results:**

All patients were followed up for 5–10 years with the mean of 6.2 ± 1.1 years. No significant differences were observed regarding the gender composition ratio, age, course of disease, number of lesion segments, and preoperative indexes of ESR, CRP, VAS score, kyphosis angle and SF-36 scale between the two groups. Besides, no significant differences were observed regarding VAS score, kyphosis angle and SF-36 scale between the two groups in the 5th postoperative year (*P* > 0.05). However, the operation time (158.2 ± 10.7 min vs. 183.7 ± 14.1 min), blood loss (517.9 ± 76.5 ml vs.714.6 ± 57.4 ml), time of ESR (2.3 ± 1.1 months vs.3.1 ± 1.4 months) and CRP (1.1 ± 0.3 months vs.1.2 ± 0.6 months) decreasing to the normal level, time of abscess disappearance (2.7 ± 1.6 months vs.4.9 ± 1.9 months), and time of bone graft fusion (6.6 ± 0.8 months vs.8.0 ± 9.6 months) in Group A were less than those in Group B (*P* < 0.05).

**Conclusions:**

Both anterior transthoracic debridement and fusion and posterior transpedicular debridement and fusion have a low risk of surgical complications and provide good quality of life for the patients with mid-thoracic (T5–9) spinal tuberculosis followed up in the mid-term. Moreover, the anterior procedure leads to early resolution of the disease and faster fusion.

## Background

Spinal tuberculosis is one of the most common extrapulmonary tuberculosis, and approximately accounts for half of bone and joint tuberculosis, which is harmful to human body and may cause vertebral collapse, kyphosis deformity and paraplegia. In recent years, with the global resurgence of tuberculosis, the incidence of spinal tuberculosis in China has been increasing year by year [1, 2]. The incidence of mid-thoracic (T5–9) spinal tuberculosis is second to that of thoracolumbar and lumbar tuberculosis, and the most vulnerable populations include young people and middle-aged people. Due to the narrow spinal canal and poor blood supply of thoracic spinal cord, the incidence of paralysis in this area is quite high once spinal canal gets involved.

The therapeutic strategy for spinal tuberculosis is based on a standard systemic anti-TB (tuberculosis) drug therapy supplemented by an appropriate surgical therapy [3, 4]. Thanks to the limited motion and stable thoracic joint support, most patients with mid-thoracic spinal tuberculosis can be treated conservatively. However, for the patients complicated with vertebral collapse, incurable abscess and severe kyphosis deformity, simple drug therapy needs a long period of medication and is prone to induce drug resistance [5–7]. Thus, it would be more appropriate to perform a surgery in combination with pharmacotherapy to remove lesions, shorten illness course, rebuild spine stability and accelerate illness recovery.

The surgical procedures for mid-thoracic spinal tuberculosis are mainly divided into anterior and posterior approaches [8, 9]. The anterior procedure includes transthoracic and extrapleural approaches, and the posterior procedure includes posterior paramedian and posterolateral approaches. Which one is chosen depends on the lesion site, abscess size, with or without spinal canal invasion, severity of spinal kyphosis, and any other factors [10, 11]. With the wide application of pedicle screws in spine operation and the rapid progress of surgical techniques, many cases treated with anterior approach only or combined anterior and posterior approach in the past can be cured with a single posterior operation today [12]. However, until now, the surgical choice for thoracic spinal tuberculosis is still controversial [13, 14]. In this study, we compared the follow-up data of the patients with mid-thoracic spinal tuberculosis who were treated with anterior transthoracic debridement and fusion with those who were treated with posterior transpedicular debridement and fusion, so as to evaluate the efficacy of the two surgical procedures.

## Methods

### Patient population

From January 2007 to June 2014, a total of 126 patients with mid-thoracic (T5–9) spinal tuberculosis who received anterior transthoracic debridement and fusion or posterior transpedicular debridement and fusion were retrospectively analyzed. This study was approved by the Ethics Review Committee of Xiangya Hospital affiliated to Central South University and all patients provided the written informed consent for the use and publication of data for research purposes. Diagnosis criteria for spinal tuberculosis include clinical presentations, such as fatigue, night sweats, low-grade fever, emaciation and back pain; imaging of bone destruction, intervertebral space narrowing, paravertebral abscess or sequestrum with X-ray, CT or MRI examinations; blood tests for erythrocyte sedimentation rate (ESR), C-reactive protein (CRP) and T cell spot tests for tuberculosis infection (T-SPOT. TB). Confirmation of TB was made using biopsy or tubercle bacillus culture ultimately.

To compare the effects between these two procedures accurately, we set up some restrictions as follows. Case inclusion criteria were as follows: (1) functional spinal units involved ≤2 segments; (2) vertebral collapse or segmental instability; (3) cavities or sequestrum appeared; (4) having complete follow-up data. Case exclusion criteria were as follows: (1) huge flow abscess; (2) active lung tuberculosis; (3) thoracic surgery history; (4) apparent presentation of spinal cord damage; (5) contraindication to surgery; (6) follow-up period < 5 years; (7) with sharp angular kyphosis. According to the above inclusion and exclusion criteria, a total of 87 cases were included in this study. Prior to the surgery, these symptoms lasted for 6–14 months.

In Group A (*n* = 39), there were 22 males and 17 females with the mean age of (35.1 ± 8.4) years (range, 20–63 years), mean disease duration of (9.6 ± 2.4) months (range, 8–17 months), mean ESR of 53.3 ± 7.7 mm/h (range, 38–78 mm/h), mean CRP of 31.9 ± 6.7 mg/l (range, 17–50 mg/l), mean VAS score of 4.7 ± 1.0 (range, 4–7), and mean kyphosis angle of 21.0° ± 5.1°(range, 17°-30°). Among them, 31 cases involved single functional spinal unit, and 8 cases involved double functional spinal units. In Group B (*n* = 48), there were 30 males and 18 females with the mean age of (37.2 ± 7.9) years (range, 26–57 years), mean disease duration of (8.9 ± 2.3) months (range, 6–14 months), mean ESR of 51.1 ± 6.8 mm/h (range, 42–65 mm/h), mean CRP of 29.6 ± 7.9 mg/l (range, 20–45 mg/l), mean VAS score of 4.4 ± 0.68 (range, 4–7), and mean kyphosis angle of 22.6° ± 3.0°(range, 20°-28°). Among them, 35 cases involved single functional spinal unit, and 13 cases involved double functional spinal units.

### Preoperative preparation

All patients received quadruple standard antituberculous chemotherapy, including oral administration of isoniazid (300 mg per day), rifampin (450 mg per day), pyrazinamide (750 mg per day) and ethambutol (750 mg per day). Anti-TB chemotherapy lasted for at least two weeks before any surgeries. It was advised that those patients who were going to receive anterior transthoracic debridement and fusion surgery should be trained on how to breathe.

### Surgical procedures

1. Anterior transthoracic debridement and fusion

**Fig. 1:**
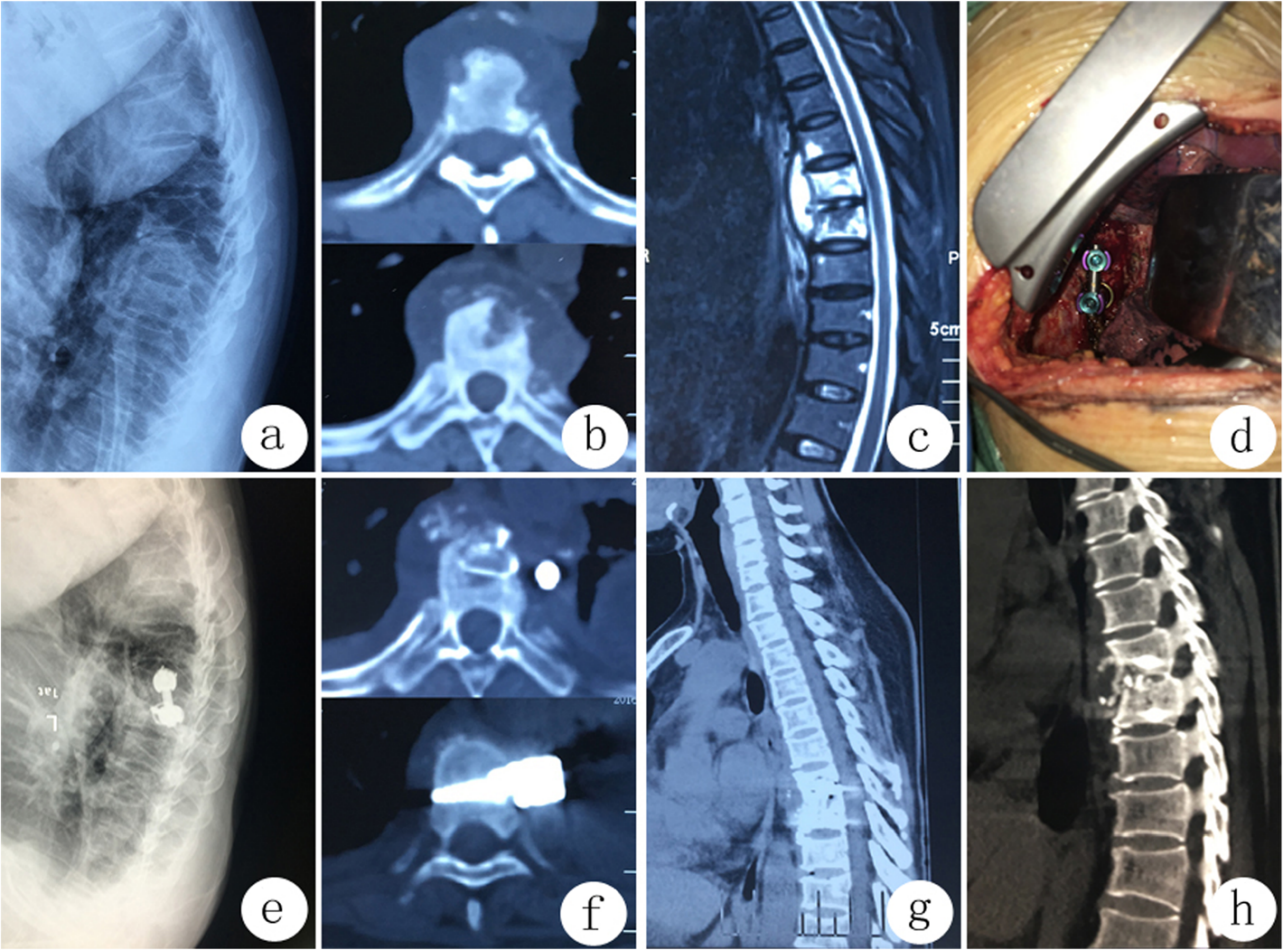
A 49-year-old female patient with T7–8 spinal tuberculosis received anterior transthoracic debridement and fusion. **1a**–**1c** Preoperative images showed paravertebral abscess formation in T7–8 and bone destruction in anterior column T8. **1d** Intraoperative findings showed that the screws and titanium rod fixation were in place after interbody grafting with direct visual observation. (1e-g). Postoperative images after two weeks of operation showed that the interbody graft and instrumentation were in place. **1 h** Postoperative images after 24 months of operation showed that solid interbody fusion was obtained

Lateral position was adopted and the lesion site should be kept upward. A cambered incision centered with the anterior-axillary line was adopted in the most serious diseased thoracic region. Subcutaneous tissue and intercostal muscle were cut layer by layer. The lung upside was collapsed for single lung ventilation. The pleural cavity was enlarged gradually using an automatic retractor without cutting ribs. Lung lobes, heart and large vessels were well protected. Abscesses, cheese-like tissue, diseased granulation tissue, necrotic discs and sequestrum were removed radically after the transverse vertebral artery was ligated securely using the LigaSure vessel-closed system. Sclerosed bones were removed using osteotome. After washing, interbody bone grafting was carried out with allograft ilium blocks. Screws were placed in the normal vertebral body adjacent to the lesion site, and titanium rods were installed and slightly pressurized to stabilize the graft. The closed drainage tube was placed for thoracic cavity and closed incision  (Fig.1).

(2) Posterior transpedicular debridement and fusion

**Fig. 2:**
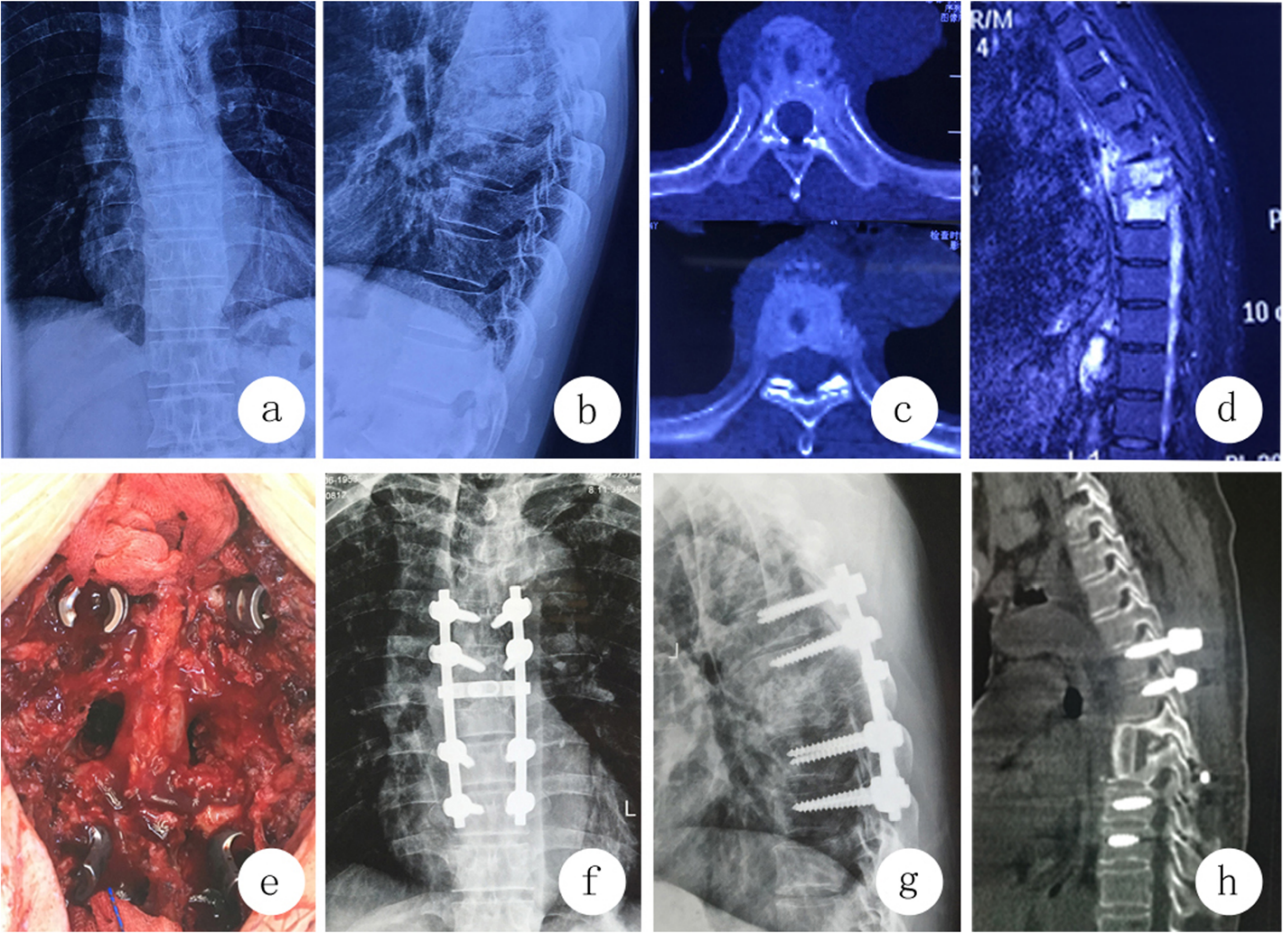
A 48-year-old female patient with T6–7 spinal tuberculosis received posterior transpedicular debridement and fusion. **2a**–**2d** Preoperative images showed paravertebral abscess formation in T6–7 and bone destruction in vertebral body of T6 and T7. **1e** Intraoperative findings showed that interbody grafting was carried out after removal of pedicles with direct visual observation. **2e**-**g** Postoperative images after two weeks of operation showed that the instrumentation was in place. **1 h** Postoperative images after 24 months of operation showed that solid interbody fusion was obtained

Patients received intubation anesthesia and a longitudinal incision was made along the spinous process of the diseased vertebrae. Subperiosteal dissection of paravertebral muscle was made to expose the lamina and zygapophyseal joints. Pedicle screws were placed in the normal vertebral body for temporary stability. Lamina, facet joints and pedicles were resected to enlarge the operating space for lesion clearance. Different curettes were used to wipe off the sequestrum and pathological granulation tissue, and osteotomes were used to remove the sclerous bone around the lesion. Anterior lesion was rinsed repeatedly. After washing, an allograft iliac bone block was implanted for interbody fusion. Titanium rods were installed for stabilization and kyphosis correction. The drainage tube was placed for wounds and the incision was closed  (Fig.2).

### Postoperative treatment

Antibiotics were used for 24–48 h. When the volume of drainage is less than 20 ml/d, the drainage tube was removed. Analgesic drug was given in time after operation. The patients who received anterior transthoracic debridement and fusion surgery were advised to carry out respiratory exercise. After the drainage removal, the patients were encouraged to get out of bed under the protection of braces. The anti-tuberculosis treatment continued for 18–20 months, during which liver function was monitored and hepatic protection treatment was provided regularly when taking anti-TB drugs. All patients were advised to put on brace apparatus for at least six months until bony fusion was observed with radiography.

### Evaluation indexes and follow-up actions

The operation duration and blood loss were recorded. The patients were followed up once a month for the first 3 months after surgery, once every 3 months for the next 9 months within the first year after surgery, once every 6 months within the second year after surgery, and once every year after 2 postoperative years. Follow-up items included blood parameters (ESR, CRP) and time of blood parameters recovering to the normal value, imaging manifestations to evaluate whether the lesion was healed or recurred, and time of bone graft fusion. Complications of perioperative and follow-up periods were also recorded. The VAS score of back pain, kyphosis angle and SF-36 scale were recorded for assessing the clinical efficacy.

### Statistics

The SPSS 22.0 (IBM, USA) statistical software was used for analysis. Comparison of the age, course of disease, operation time, blood loss, number of functional spinal units involved, ESR, CRP and time of ESR and CRP recovery to the normal value, time of abscess disappearance, time of bone graft fusion, VAS score, kyphosis angle and SF-36 scale were used for the independent-samples *t* test. The gender composition ratio and incidence of complications were compared through the chi-square test, and *P* value less than 0.05 on both sides was considered to be significantly different.

## Results

All patients were followed up for 5–10 years with the mean of 6.2 ± 1.1 years. No significant differences were observed regarding the gender composition ratio, age, course of disease, number of lesion segments, and preoperative indexes of ESR, CRP, VAS, kyphosis angle and SF-36 score between the two groups (Tables 1, 2 and 3). The mean operation time was 158.2 ± 10.7 min and the mean blood loss was 517.9 ± 76.5 ml in Group A, both of which were significantly less than those in Group B (183.7 ± 14.1 min and 714.6 ± 57.4 ml) (*P* < 0.05) (Table 1). The time of ESR and CRP decreasing to normal level were 2.3 ± 1.1 and 1.1 ± 0.3 months respectively in Group A, both of which were significantly less than those in Group B (3.1 ± 1.4 and 1.2 ± 0.6 months) (*P* < 0.05) (Table 2). The time of abscess disappearance and bone graft fusion were 2.7 ± 1.6 and 6.6 ± 0.8 months respectively, both of which were significantly less than those in Group B (4.9 ± 1.9 and 8.0 ± 9.6 months) (Table 2). In Group A, severe intercostal neuralgia occurred in 3 cases, and ultimately was healed through nerve blocks; after that, no other major complications occurred. In Group B, cerebrospinal fluid leakage occurred in 2 cases, and resurgence occurred in 4 cases and ultimately was cured via abscess drainage under thoracoscope surveillance, and superficial wound infection occurred in 1 case. No neurological, vascular or other major complications were found during the operation and follow-up in the two groups. The rate of surgical complications was 7.7% (3/39) and 14.6% (7/48) respectively in Group A and B, the difference in the rate of complications between the two groups was not significant (*P* > 0.05) (Table 2). In the 5th postoperative year, there were no significant difference regarding VAS score, kyphosis angle and SF-36 scale between the two groups (*P* > 0.05) (Tables 2, 3).

**Table 1:** Basic clinical data of patients

	Group A (*N* = 39)	Group B (*N* = 48)	*P* Value
Gender (male/female)	22/17	30/18	0.565
Age (years)	35.1 ± 8.4	37.2 ± 7.9	0.242
Disease duration (months)	9.6 ± 2.4	8.9 ± 2.3	0.234
Number of affected segments	1.2 ± 0.41	1.3 ± 0.45	0.482
Pre-ESR (mm/h)	53.3 ± 7.7	51.1 ± 6.8	0.153
Pre-CRP (mg/L)	31.9 ± 6.7	29.6 ± 7.9	0.149
Pre-VAS score	4.7 ± 1.0	4.4 ± 0.68	0.082
Pre-Kyphosis angle	21.0 ± 5.1	22.6 ± 3.0	0.09

Data are presented as *n* (%) or mean ± standard deviation (range)

**Table 2:** Evaluation indexes Comparison between Group A and Group B

Evaluation indexes	Group A (N = 39)	Group B (N = 48)	*P* Value
Operation time (min)	158.2 ± 10.7	183.7 ± 14.1	0.000
Blood loss (ml)	517.9 ± 76.5	714.6 ± 57.4	0.000
Time of ESR descending to normal (months)	2.3 ± 1.1	3.1 ± 1.4	0.000
Time of CRP descending to normal (months)	1.1 ± 0.3	1.2 ± 0.6	0.000
Time of abscess disappearance (months)	2.7 ± 1.6	4.9 ± 1.9	0.000
Time of bone graft fusion (months)	6.6 ± 0.8	8.0 ± 9.6	0.000
Rate of surgical complications(%)	7.7 (3/39)	14.6 (7/48)	0.370
VAS score in postoperative 5th year	1.0 ± 0.6	1.1 ± 0.4	0.449
Kyphosis angle in postoperative 5th year	9.9 ± 2.1	10.3 ± 1.9	0.266

Data are presented as n (%) or mean ± standard deviation (range)

**Table 3:** SF-36 Scores of Comparison between Group A and Group B

		Group A	Group B	*P* Value
Pre-op	PF	64.0 ± 5.3	65.4 ± 6.3	0.259
	RP	14.7 ± 13.7	15.1 ± 16.1	0.912
	BP	43.5 ± 8.6	41.6 ± 9.0	0.305
	GH	26.4 ± 14.6	24.6 ± 11.9	0.538
	VT	45.6 ± 11.0	44.8 ± 11.7	0.729
	SF	27.9 ± 9.3	26.6 ± 8.0	0.495
	RE	35.9 ± 16.0	34.0 ± 16.1	0.591
	MH	29.5 ± 11.5	31.0 ± 11.5	0.532
Post-op 5th Year	PF*	81.1 ± 5.9	82.9 ± 5.6	0.152
	RP*	88.0 ± 15.5	92.3 ± 14.2	0.186
	BP*	86.0 ± 6.6	87.2 ± 7.2	0.420
	GH*	80.0 ± 12.5	81.8 ± 13.3	0504
	VT*	88.1 ± 6.1	78.8 ± 8.7	0.211
	SF*	76.6 ± 7.6	86.6 ± 5.1	0.194
	RE*	88.8 ± 15.9	89.7 ± 15.6	0.802
	MH*	82.1 ± 9.4	84.1 ± 11.6	0.374

*PF* Physical Functioning, *RP* Role-Physical, *BP* Bodily Pain, *GH* General Health, *VT* Vitality, *SF* Social Functioning, *RE* Role- Emotional, *MH* Mental Health

*Pre-op* pre-operation, *Post-op* post-operation

^*^: *P* < 0.05, compared with pre-op indexes

## Discussions

Anti-TB drug therapy plays an important role in treating spinal tuberculosis because drug therapy is the basis for surgical treatment. In this study, the preoperative and postoperative normative anti-TB treatments were carried out in all cases for 2 weeks and 18–20 months respectively. Lesion clearance, bone grafting and internal fixation are three key techniques for the surgical treatment of spinal tuberculosis. Lesion clearance is the cornerstone for bone grafting and internal fixation, so it is dangerous to ignore lesion clearance and pursue bone grafting and internal fixation blindly [15, 16].

In our series, there were no recurrent or uncured cases in the anterior debridement and fusion group; 4 uncured cases were found in the posterior transpedicular debridement and fusion group initially, and were then cured after a second anterior procedure of abscess evacuation with the help of thoracoscope. In the 5th postoperative years, all ESR and CRP decreased into the normal range, and all paravertebral abscesses disappeared and all bone grafting was fused in the two groups. Meanwhile, similar clinical results regarding VAS score, kyphosis angle and SF-36 scale were observed in the two groups in the 5th postoperative year. So, our research indicates that satisfactory clinical, radiological and functional results were obtained from the mid-term follow-up for both anterior transthoracic debridement and fusion and posterior transpedicular debridement and fusion.

Because most spinal tuberculosis lesions are located in the anterior and middle column of the spine and there is no obstruction of the scapula or diaphragm muscle in the mid-thoracic region, adopting anterior transthoracic debridement and fusion for mid-thoracic spinal tuberculosis has an innate advantage over other approaches and surgeons dealing with bone grafting and fixation simultaneously after radical debridement [17, 18]. Dai et al. [19] conducted a prospective study of 39 cases with spinal tuberculosis, who were going to receive single-stage anterior debridement, bone grafting and instrumentation. They found that the therapeutic effect of the single anterior approach was excellent with a very low unhealing rate. Hassan et al. [20] compared 42 cases of anterior versus posterior approach for the treatment of thoracic and lumbar tuberculous spondylodiscitis, and they found that both approaches were effective for the treatment of thoracic and lumbar tuberculosis. Jin et al. [21] applied the anterior approach to 21 cases of adults with thoracolumbar tuberculosis and 2 cases of children with thoracolumbar tuberculosis, and they found that anterior interbody autograft and instrumentation were effective for adults, but poor for children in maintaining kyphosis correction. Lü et al. [22] reported that 50 cases with thoracic spinal tuberculosis received thoracoscopy-assisted anterior debridement and reconstruction, and it was found that the clinical and radiographic outcomes for the minimal invasive anterior procedure were exciting, but a long learning cure was required to grasp the operational technique. However, the anterior approach also has its inherent disadvantages, for example, the strength of vertebral body fixation is poorer than that of pedicle screws, and surgeons are required to familiarize with the anatomy of thoracic cavity and have skillful thoracic surgery techniques [23].

Some scholars believe that the anterior approach is more traumatic because it always needs resecting part of the ribs and disturbing lungs and large vessels. Wang et al. [24] reported that the single anterior approach needed a longer time and more blood loss than the single posterior approach. Wu et al. [13] conducted a multi-center retrospective study for patients with thoracic spinal tuberculosis, and it was found that anterior surgeries can achieve similar efficacy as posterior-only surgeries, but with more trauma, more blood loss and shorter operation time. However, Cui et al. [25] reported that anterior surgeries consume less operation time and are less traumatic than posterior surgeries. Dunn et al. [1] deemed that the anterior approach is more suitable for thoracic spinal tuberculosis because with transthoracic approach, debridement is easier and more effective without sacrificing nerve roots.

In our series, we found that the operation time and blood loss of anterior operations are significantly lower than those of posterior operations. It means that anterior transthoracic debridement and fusion is less traumatic than posterior transpedicular debridement and fusion, which is contrary to some of the previous studies [13, 24]. The observation may be explained in several aspects. Firstly, the focal area involved in this study is located in the mid-thoracic region and the affected functional spinal units are composed of no more than 2 segments, so good lesion exposure and a convenient operating space could be obtained without cutting off the ribs. Secondly, how vertebral transverse arteries are managed imposes an important impact on surgical trauma. In the past, we had to isolate and ligate vertebral transverse arteries with silk suture lines. Due to the deep location of vertebral transverse artery in the thoracic cavity, ligation was always difficult and prone to slipping away. In this study, we used the LigaSure vessel-closed system to manage vertebral transverse arteries with less time and less blood loss. Thirdly, in this study, surgeons from the Thoracic Surgery Department were required to cooperate with all patients treated with anterior transthoracic debridement and fusion during the operation. In our study, the time of inflammatory biomarkers decreasing to the normal level, abscess disappearance and bone graft fusion in the anterior group were significantly less than those in the posterior group, which indicates anterior transthoracic debridement and fusion not only causes less trauma than posterior transthoracic debridement and fusion, but also allows for faster patient recovery.

Some experts are concerned about the safety of the anterior approach due to the presence of heart, lungs, large blood vessels and other important tissues in the thoracic cavity, as even a tiny careless mistake can be life-threatening. However, both previous studies and our study have shown that the mortality and rate of major complications were very low [26, 27]. Some scholars suspected that posterior transpedicular debridement and fusion might cause tuberculosis spreading and normal structure destruction in the posterior column. However, a number of literatures [28–30] and our study have proven the safety of the procedure. In this study, we have found that both anterior surgeries and posterior surgeries have low rates of complications, which is in line with the results of most of the previous studies.

This study is a retrospective study, so there are some inherent limitations, such study design, recall bias, selection bias, missing information and some other subjective factors. In addition, the position of the infection, degree of debridement, and alignment change are all important factors for surgical approach deciding in clinical works, although we made some restrictions for cases selection and did some statistical analysis to minimize the interference on the results. This is a single center study and the number of cases is small, so the conclusion need to be further verified in multi-center prospective randomized controlled study.

## Conclusions

Both anterior transthoracic debridement and fusion and posterior transpedicular debridement and fusion have a low risk of surgical complications and provide good quality of life for the patients with mid-thoracic (T5–9) spinal tuberculosis followed up in the mid-term. However, anterior transthoracic debridement and fusion was less traumatic and allows for faster patient recovery.

## Data Availability

The datasets supporting the conclusions of this article are included within the article. The raw data can be requested from the corresponding author on reasonable request.

## Abbreviations

BP: Bodily Pain
CRP: C-reactive protein
ESR: Erythrocyte Sedimentation Rate
GH: General Health
MH: Mental Health
PF: Physical Functioning
Pre-CRP: Preoperative CRP
Pre-ESR: Preoperative ESR
Pre-op: Preoperation; Post-op: Postoperation
Pre-VAS: Preoperative VAS
RE: Role-Emotional
RP: Role-Physical
SF: Social Functioning
SF-36: Social Functioning-36
TB: Tuberculosis
T-SPOT: TB: T cell spot tests for tuberculosis infection
VT: Vitality

## References

[1] Dunn RN, Ben HM (2018). Spinal tuberculosis. Bone Joint J..

[2] Thakur K, Das M, Dooley KE (2018). The Global Neurological Burden of Tuberculosis. Semin Neurol.

[3] Nene A, Bhojraj S (2005). Results of nonsurgical treatment of thoracic spinal tuberculosis in adults. Spine J.

[4] Bodapati PC, Vemula RCV, Mohammad AA (2017). Outcome and management of spinal tuberculosis according to severity at a tertiary referral center. Asian J Neurosurg.

[5] Wang Z, Shi J, Geng G (2013). Ultra-short-course chemotherapy for spinal tuberculosis: five years of observation. Eur Spine J.

[6] Martín-Alonso J, Delgado-López PD, Castilla-Díez JM (2018). Role of surgery in spontaneous spondylodiscitis: experience in 83 consecutive patients. Neurocirugia (Astur).

[7] Soares Do Brito J, Tirado A, Fernandes P (2014). Surgical treatment of spinal tuberculosis complicated with extensive abscess. Iowa Orthop J.

[8] Hodgson AR, Stock FE (1956). Anterior spinal fusion a preliminary communication on the radical treatment of Pott’s disease and Pott’s paraplegia. Br J Surg.

[9] Luo C, Wang X, Wu P (2016). Single-stage transpedicular decompression, debridement, posterior instrumentation, and fusion for thoracic tuberculosis with kyphosis and spinal cord compression in aged individuals. Spine J.

[10] Chandra SP, Singh A, Goyal N (2013). Analysis of changing paradigms of management in 179 patients with spinal tuberculosis over a 12-year period and proposal of a new management algorithm. World Neurosurg.

[11] Kumar MN, Joseph B, Manur R (2013). Isolated posterior instrumentation for selected cases of thoraco-lumbar spinal tuberculosis without anterior instrumentation and without anterior or posterior bone grafting. Eur Spine J.

[12] Li L, Xu J, Ma Y (2014). Surgical strategy and management outcomes for adjacent multisegmental spinal tuberculosis: a retrospective study of forty-eight patients. Spine (Phila Pa 1976).

[13] Wu W, Lyu J, Liu X (2018). Surgical treatment of thoracic spinal tuberculosis: a multicenter retrospective study. World Neurosurg..

[14] Meena S, Mittal S, Chowdhary B (2014). Spinal tuberculosis: which is the best surgical approach?. Med Princ Pract.

[15] Jin W, Wang Q, Wang Z, Geng G (2014). Complete debridement for treatment of thoracolumbar spinal tuberculosis: a clinical curative effect observation. Spine J.

[16] Wang B, Kong L, Zhu Z (2018). Recurrent complex spinal tuberculosis accompanied by sinus tract formation: causes of recurrence and clinical treatments. Sci Rep.

[17] Wang ST, Ma HL, Lin CP (2016). Anterior debridement may not be necessary in the treatment of tuberculous spondylitis of the thoracic and lumbar spine in adults: a retrospective study. Bone Joint J..

[18] Wang Z, Ge Z, Jin W (2007). Treatment of spinal tuberculosis with ultrashort-course chemotherapy in conjunction with partial excision of pathologic vertebrae. Spine J.

[19] Dai LY, Jiang LS, Wang W (2005). Single-stage anterior autogenous bone grafting and instrumentation in the surgical management of spinal tuberculosis. Spine (Phila Pa 1976).

[20] Hassan K, Elmorshidy E (2016). Anterior versus posterior approach in surgical treatment of tuberculous spondylodiscitis of thoracic and lumbar spine. Eur Spine J.

[21] Jin D, Qu D, Chen J (2004). One-stage anterior interbody autografting and instrumentation in primary surgical management of thoracolumbar spinal tuberculosis. Eur Spine J.

[22] Lü G, Wang B, Li J (2012). Anterior debridement and reconstruction via thoracoscopy-assisted mini-open approach for the treatment of thoracic spinal tuberculosis: minimum 5-year follow-up. Eur Spine J.

[23] Shi J, Tang X, Xu Y (2014). Single-stage internal fixation for thoracolumbar spinal tuberculosis using 4 different surgical approaches. J Spinal Disord Tech.

[24] Wang LJ, Zhang HQ, Tang MX (2017). Comparison of three surgical approaches for thoracic spinal tuberculosis in adult: minimum 5-year follow up. Spine (Phila Pa 1976).

[25] Cui X, Li LT, Ma YZ (2016). Anterior and posterior instrumentation with different debridement and grafting procedures for multi-level contiguous thoracic spinal tuberculosis. Orthop Surg.

[26] Li M, Du J, Meng H (2011). One-stage surgical management for thoracic tuberculosis by anterior debridement, decompression and autogenous rib grafts, and instrumentation. Spine J.

[27] Benli IT, Kaya A, Acaroğlu E (2007). Anterior instrumentation in tuberculous spondylitis: is it effective and safe?. Clin Orthop Relat Res.

[28] Wang X, Pang X, Wu P (2014). One-stage anterior debridement, bone grafting and posterior instrumentation vs. single posterior debridement, bone grafting, and instrumentation for the treatment of thoracic and lumbar spinal tuberculosis. Eur Spine J.

[29] Liu Z, Zhang P, Zeng H (2018). A comparative study of single-stage transpedicular debridement, fusion, and posterior long-segment versus short-segment fixation for the treatment of thoracolumbar spinal tuberculosis in adults: minimum five year follow-up outcomes. Int Orthop.

[30] Ukunda UNF, Lukhele MM (2018). The posterior-only surgical approach in the treatment of tuberculosis of the spine. Bone Joint J.

